# Host–guest interactions in *nor*-*seco*-cucurbit[10]uril: novel guest-dependent molecular recognition and stereoisomerism

**DOI:** 10.3762/bjoc.15.166

**Published:** 2019-07-19

**Authors:** Xiaodong Zhang, Wei Wu, Zhu Tao, Xin-Long Ni

**Affiliations:** 1 Key Laboratory of Macrocyclic and Supramolecular Chemistry of Guizhou Province, Guizhou University, Guiyang 550025, China

**Keywords:** fluorescent, host–guest interaction, macrocycles, molecular recognition, *nor*-*seco*-cucurbit[10]uril, pyrene

## Abstract

The unique monomer and excimer fluorescence emissions of pyrene were first exploited as distinctly photophysical signals to identify the possible diastereomers of guests within *nor*-*seco*-cucurbit[10]uril (NS-CB[10]) cavities. Further experiments revealed that balancing the hydrophilic and hydrophobic effects of the guest in aqueous solution can improve the molecular recognition and binding ability of NS-CB[10].

## Introduction

Host–guest interactions that trigger molecular recognition are a current topic of interest. For example, understanding the protein–ligand molecular recognition is of paramount importance in the study of enzymatic catalysis and allosteric regulation of cell signaling, as well as in the design of efficient drugs that utilize host–guest interactions [1]. Cucurbit[*n*]urils (Q[*n*]s or CB[*n*]s) [2–3] having been viewed to have high potential use in host–guest chemistry in aqueous solution because of their varying cavity rigidity and larger portal sizes as compared with those of other macrocyclic hosts [4–13]. For example, cucurbit[8]uril (Q[8] or CB[8]), a large homologue of the Q[*n*] family, is unique because of its ability to bind two hetero- and homo-aromatic guests in its cavity through host-stabilized charge-transfer or π–π interactions [14–15]. This novel property of Q[8] has been utilized as molecular container for biological substrates [16–17], as well as in the construction of various supramolecular assemblies with specific structures and properties [18–22]. However, forming ternary complexes with Q[8] is challenging because the number of aromatic-derived water-soluble recognition motifs remains limited.

**Scheme 1:**
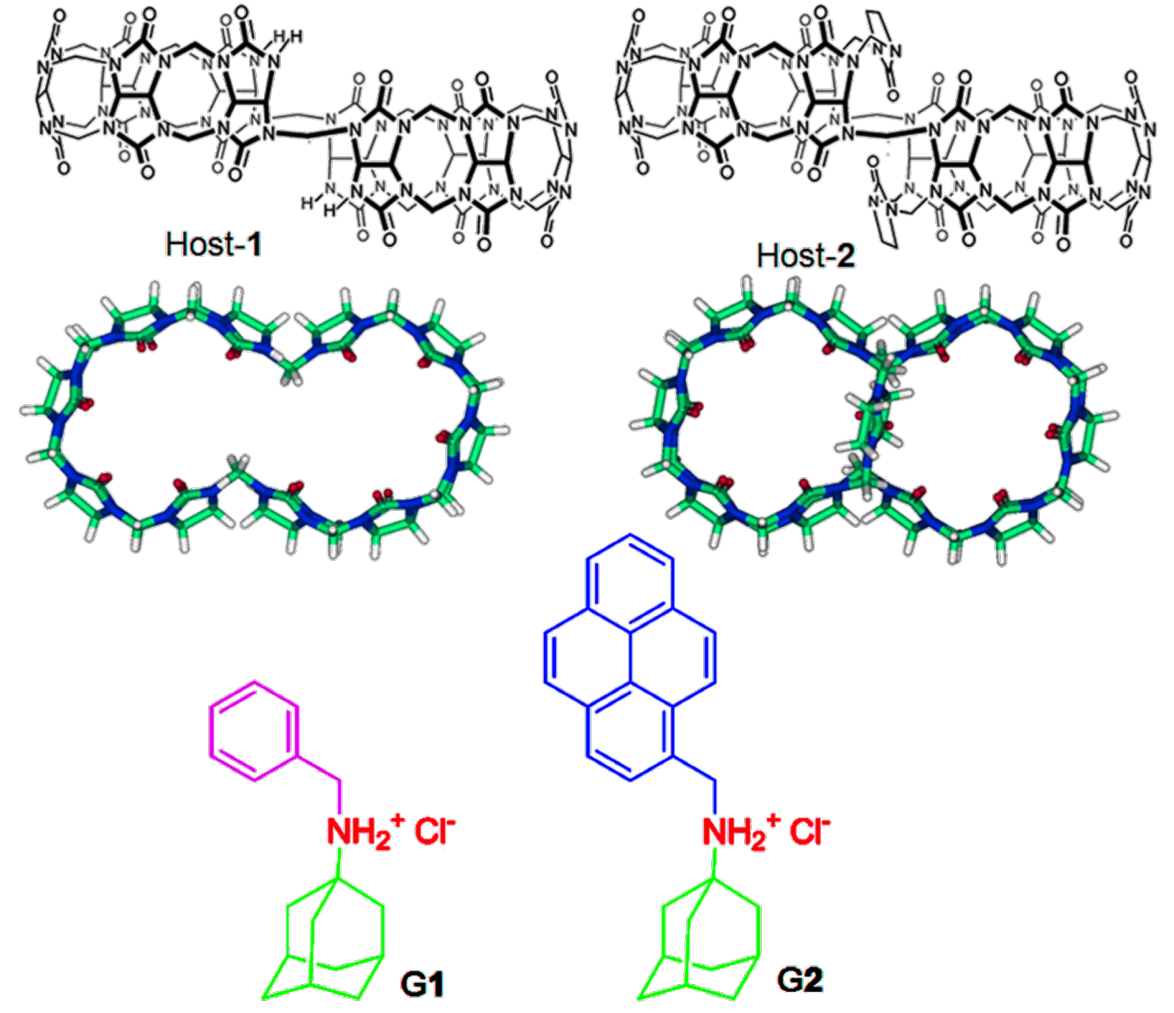
Chemical structures of host-**1**, host-**2**, **G1**, and **G2**.

In 2006, Isaacs and co-workers reported the synthesis and isolation of *nor*-*seco*-cucurbit[10]uril (NS-CB[10], host-**1**, Scheme 1) [23], a new member of the extended Q[*n*] family that contains two identical cavities. Different from the Q[8] host, NS-CB[10] can not only accommodate two aromatic guest molecules such as 4,4-bipyridinium (viologen), but also has the ability to accommodate two other guest molecules such as adamantaneammonium (ADA) or alkylammonium ions into the cavity, forming a ternary complex. The novel binding capacity of NS-CB[10] has been utilized to form supramolecular polymers [24–26] and polymer nanoparticles [27]. More importantly, Isaacs et al. discovered that when the unsymmetrical guest ADA molecules are bound within NS-CB[10], three diastereomers such as top–top, center–center, and top–center can be observed. These diastereomers display homotropic allostery based on a guest-size-induced preorganization mechanism [23]. However, the same ADA guest was not incorporated into the cavity after NS-CB[10] was functionalized with imidazolidone (host-**2**, Scheme 1) [28].

Herein, we report the supramolecular host–guest interactions of the two cavities of NS-CB[10]-based host*-***1** and host**-2** with two unsymmetrical ADA-based derivatives (**G1** and **G2**, Scheme 1). As expected, hosts**-1** and -**2** are capable of simultaneously binding guest **G1**, thus forming 1:2 ternary complexes by including different groups of **G1** into the cavity. For example, host-**1** can accommodate the ADA moiety into the cavity, whereas host**-2** tends to include the benzyl group into the cavity. This behavior may be attributed to the bridging imidazolidone units of host**-2** that rigidify its structure and make it selective toward smaller guests. However, we found that the ADA group can be accommodated into the cavity of host-**2** when a larger hydrophobic unit such as pyrene instead of a benzyl group was appended to the ADA scaffold (**G2**). Interestingly, the top–center isomer of **G2** within both host-**1** and host-**2** could be characterized because of the novel monomer and excimer photophysical property of pyrene as fluorophore. As a result, we demonstrated here a novel guest-controlled molecular recognition and stereoisomerism for the first time.

## Results and Discussion

**Figure 1:**
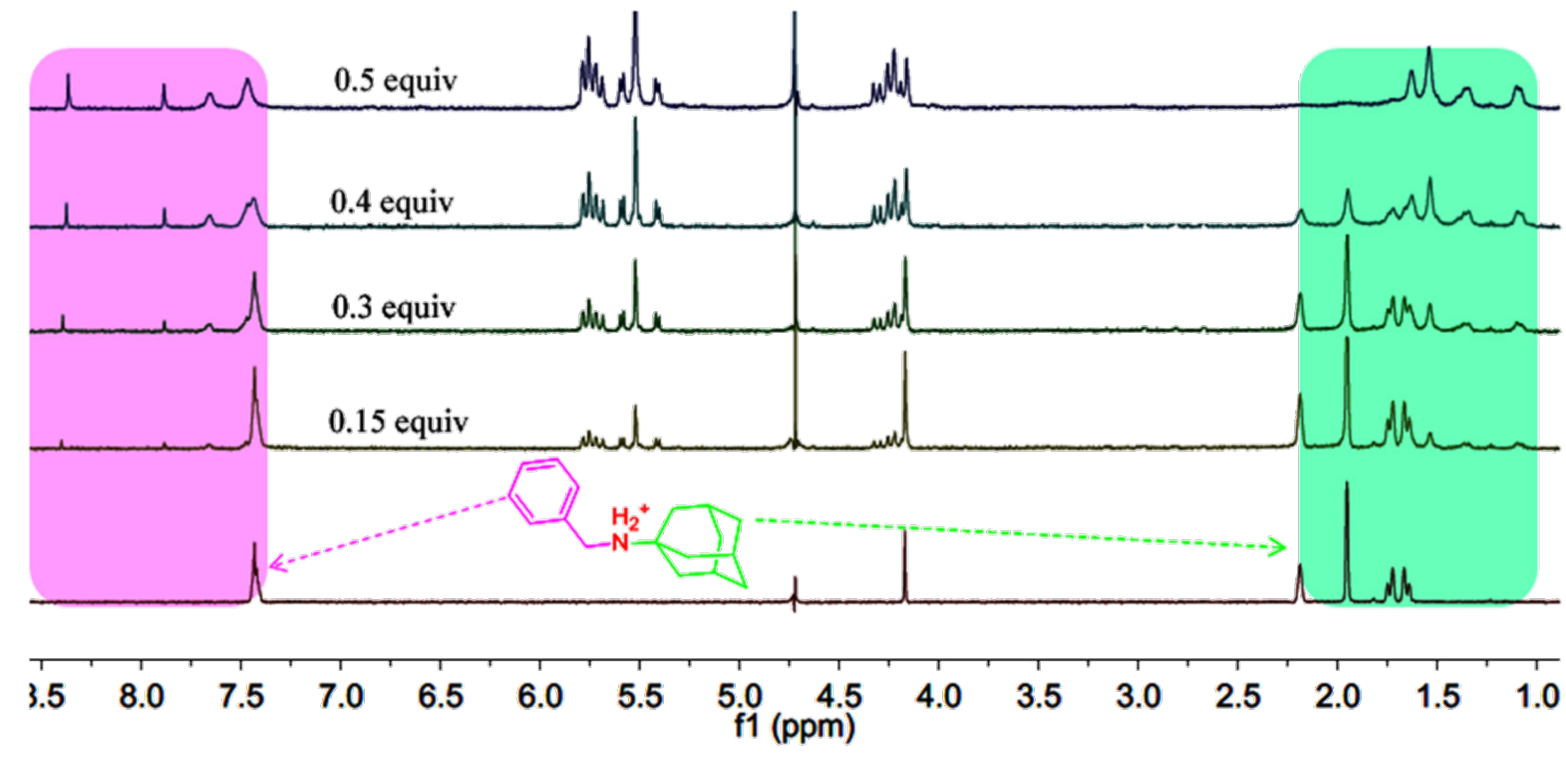
^1^H NMR spectra of **G1** (1.0 mmol, D_2_O, pD = 2.0) in the presence of host-**1** at different concentrations at 298K.

We took advantage of the two novel identical cavities and the simple formation of ternary diastereomer complexes with NS-CB[10]-based host-**1** and host-**2**. The size of each cavity of host-**1** is similar in size to Q[7], and the cavity size of host-**2** is close to that of Q[6]. We first designed and prepared the ADA-benzyl-based ammonium guest molecules **G1**. We synthesized this compound because ADA can form a highly stable complex with Q[7] (the highest reported *K* value for the ADA·Q[7] complex is 10^12^ M^−1^) [29]; while benzylammonium ions can be included in both cavities of Q[7] and Q[6]. Figure 1 shows the ^1^H NMR spectral changes of the **G1** guest in D_2_O (pD = 2.0) in the presence of host**-1** at different concentrations. Upon gradual addition of host**-1** (0–0.4 equiv) to the solution of **G1**, the resonances corresponding to the protons on **G1** split into two sets of signals. For example, one shifted upfield (ADA moiety) or downfield (benzyl moiety), and one remained at the original position. This result may be attributed to the slow movements of complexed and uncomplexed forms of **G1** with host**-1** on the NMR time scale; therefore, free **G1** and the **G1**·host**-1** complex were individually observed. With increasing concentrations of host-**1** to ca. 0.5 equiv (Figure 1), the original proton signal disappeared. In particular, the peak of the protons on the ADA moiety shifted upfield from δ 2.23–1.65 ppm to δ 1.60–1.09 ppm, whereas the signals for the protons on the benzyl group substantially shifted downfield from δ 7.44 ppm to 7.48, 7.65, 7.89, and 8.38 ppm. These results suggest that host-**1** cavities encapsulated the ADA moiety of **G1** (upfield shift due to the shielding effect of the hydrophobic cavity) and that the benzyl group was on or near the host-**1** portal (downfield shift due to the deshielding effect of the carbonyl-rimmed portal). Isothermal titration calorimetry results reveal a 2:1 **G1**/host**-1** stoichiometry with a binding constant (*K*_a_) of 1.32 × 10^5^ M^−1^ (Table 1). These observations clearly indicate that host-**1** recognizes and prefers to include the ADA moieties in both of the identical cavities with homotropic allostery effect.

**Figure 2:**
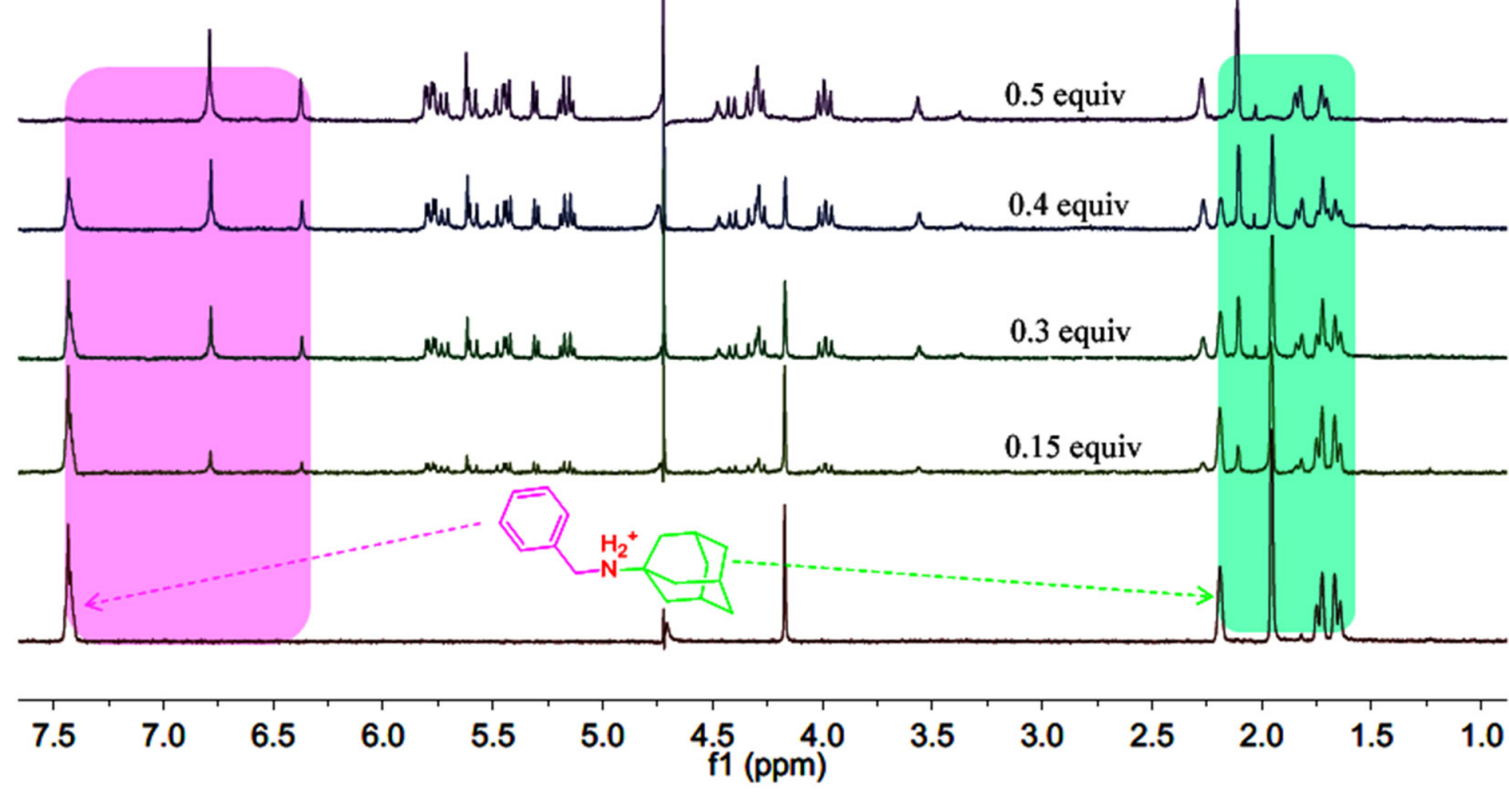
^1^H NMR spectra of **G1** (1.0 mmol, D_2_O, pD = 2.0) in the presence of host-**2** at different concentrations at 298 K.

In the case of the ^1^H NMR titration experiments for host–guest interactions of **G1**·host**-2**, the chemical shift changes in **G1** with increasing host-**2** concentration are similar to those observed in the host**-1** systems. However, the largest difference in the host–guest interaction properties between **G1·**host**-2** and **G1·**host**-1** is that the proton peaks on the benzyl group of **G1** undergoes a large upfield shift (from δ 7.44 ppm to 6.75 and 6.37 ppm), as well as a slight proton downfield shift of the ADA moiety in the presence of host**-2** (Figure 2). These spectral changes suggest that **G1** accommodated its benzyl group into the cavities and that the ADA moiety remained in the portal of host**-2**, forming ternary complexes. These findings are also consistent with the behaviors observed by Isaacs and co-workers. That is, the bridging imidazolidone units of host**-2** rigidify the structure of host**-1**, which has the ability to expand its cavity to accommodate larger guests. This effect makes it selective toward small guests [12].

As mentioned previously, Isaacs and co-workers reported their pioneering work on the host–guest interaction properties of host-**1** and -**2**. Several diastereomers of some guests such as ADA were observed within ternary complexes of host-**1** by ^1^H NMR spectroscopy [23]. However, when we appended the benzyl group to the ADA moiety (**G1**) in the present study, the ^1^H NMR spectra cannot provide the related proton signals that distinguish the possible diastereomers of **G1** in host-**1** and host-**2**, despite the clear split of resonances corresponding to the protons on the hosts.

**Scheme 2:**
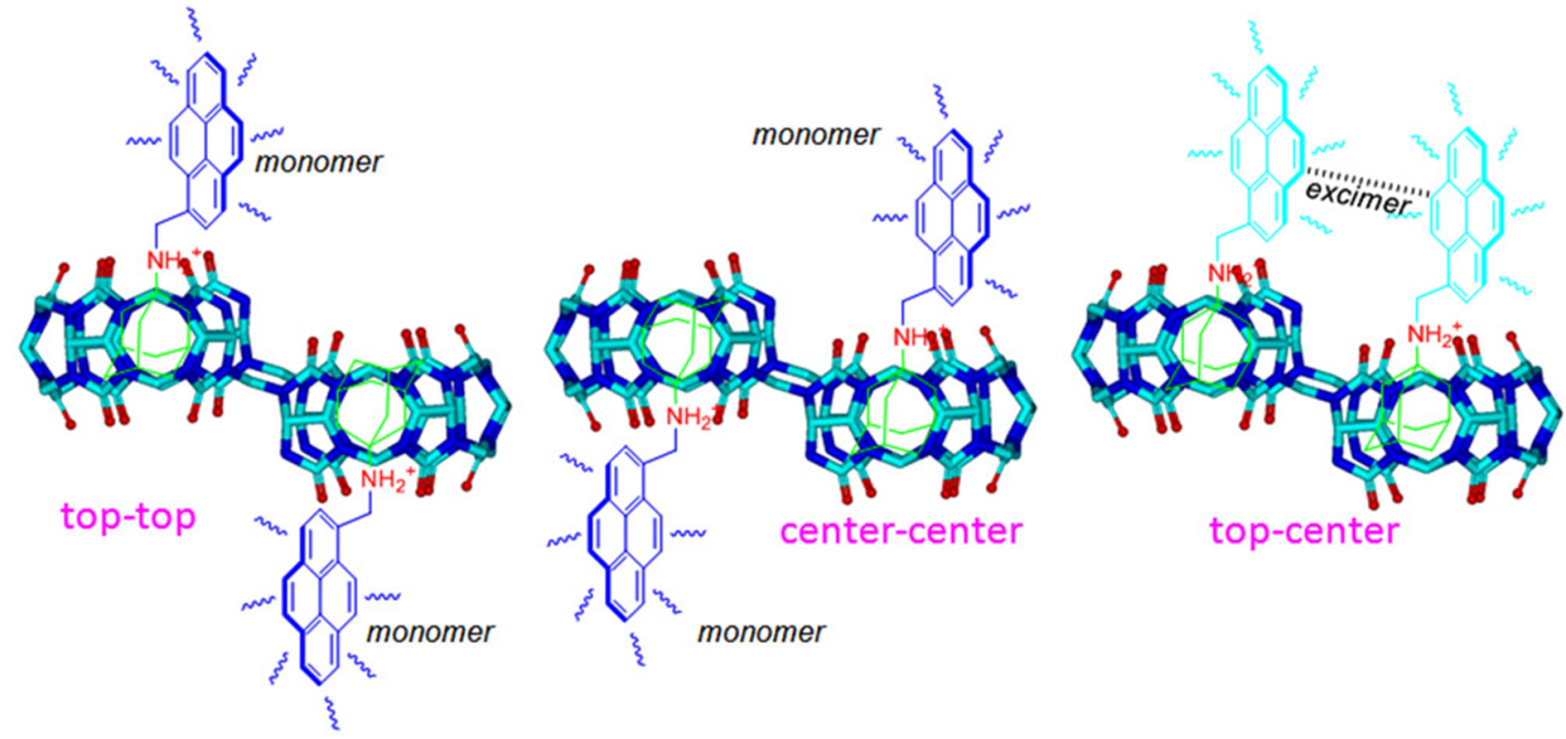
Plausible diastereomers showing the fluorescence response of **G2** with host-**1**.

A number of studies suggest that pyrene is one of the most useful fluorogenic units, being sensitive to conformational change because of its relatively efficient monomer and excimer emissions [30]. For example, when pyrene instead of the benzyl group was appended to the ADA moiety (**G2**) in the present work, the top–center diastereomers of ADA-based derivatives within the host cavities could be distinguished by the novel photophysical property of pyrene (Scheme 2).

**Figure 3:**
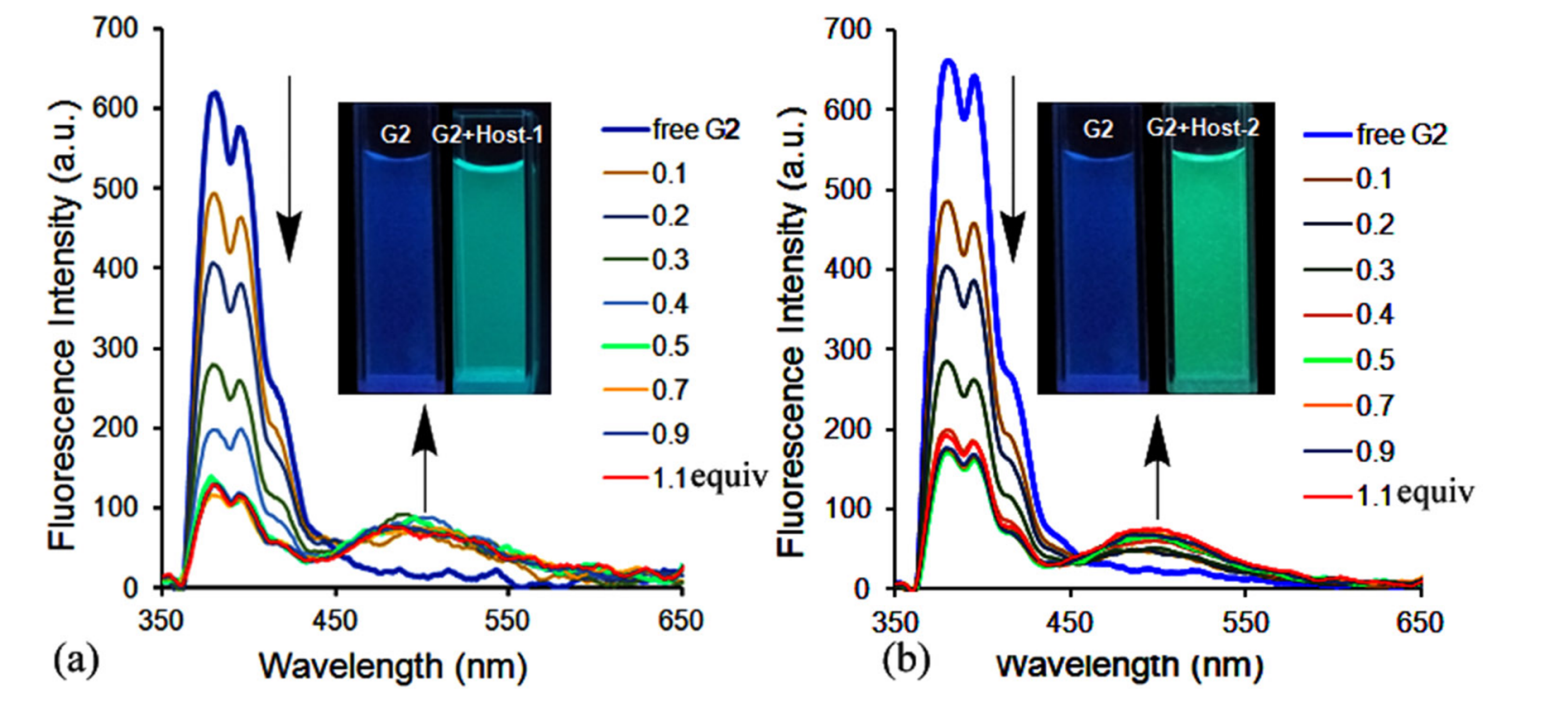
Fluorescence spectral changes of **G2** (10.0 μM) in the presence of host-**1** (a) and host-**2** (b) at different concentrations in aqueous solution (pH 2.0) at 298 K.

Figure 3a shows changes in the fluorescence emission spectra of **G2** in the presence of host-**1**. As can be seen, free **G2** produced typical monomer emissions at around 378 and 396 nm in aqueous solution (pH 2) upon excitation of the pyrene fluorophore at 340 nm. When we added host**-1** at increasing concentrations to the **G2** solution, the fluorescence intensity of the **G2** monomer emissions gradually decreased, while the maximum emission intensity at around 485 nm (typical excimer emissions of pyrene) increased. The excimer emission band of **G2** can be attributed to the interaction of two pyrene units resulting in intermolecular π–π stacking, which was due to the two identical cavities of host-**1**. Consequently, the top–center isomerism of the **G2·**host-**1** ternary complexes was conveniently confirmed from the optical signal after the changes in monomer/excimer fluorescence emissions of the pyrene groups on **G2**. Surprisingly, the fluorescence spectral changes of **G2** (Figure 3b) suggest that a similar host–guest interaction triggered monomer-to-excimer binding response between **G2** and host **2**, similar to host **1** with **G2**, when we attempted to add host**-2** to the **G2** solution under the same conditions. Both of the complexes of **G2** with the host-**1** ad host-**2** were further studied by UV–vis spectra (Supporting Information File 1, Figure S3) and Job plot (Supporting Information File 1, Figure S4).

**Figure 4:**
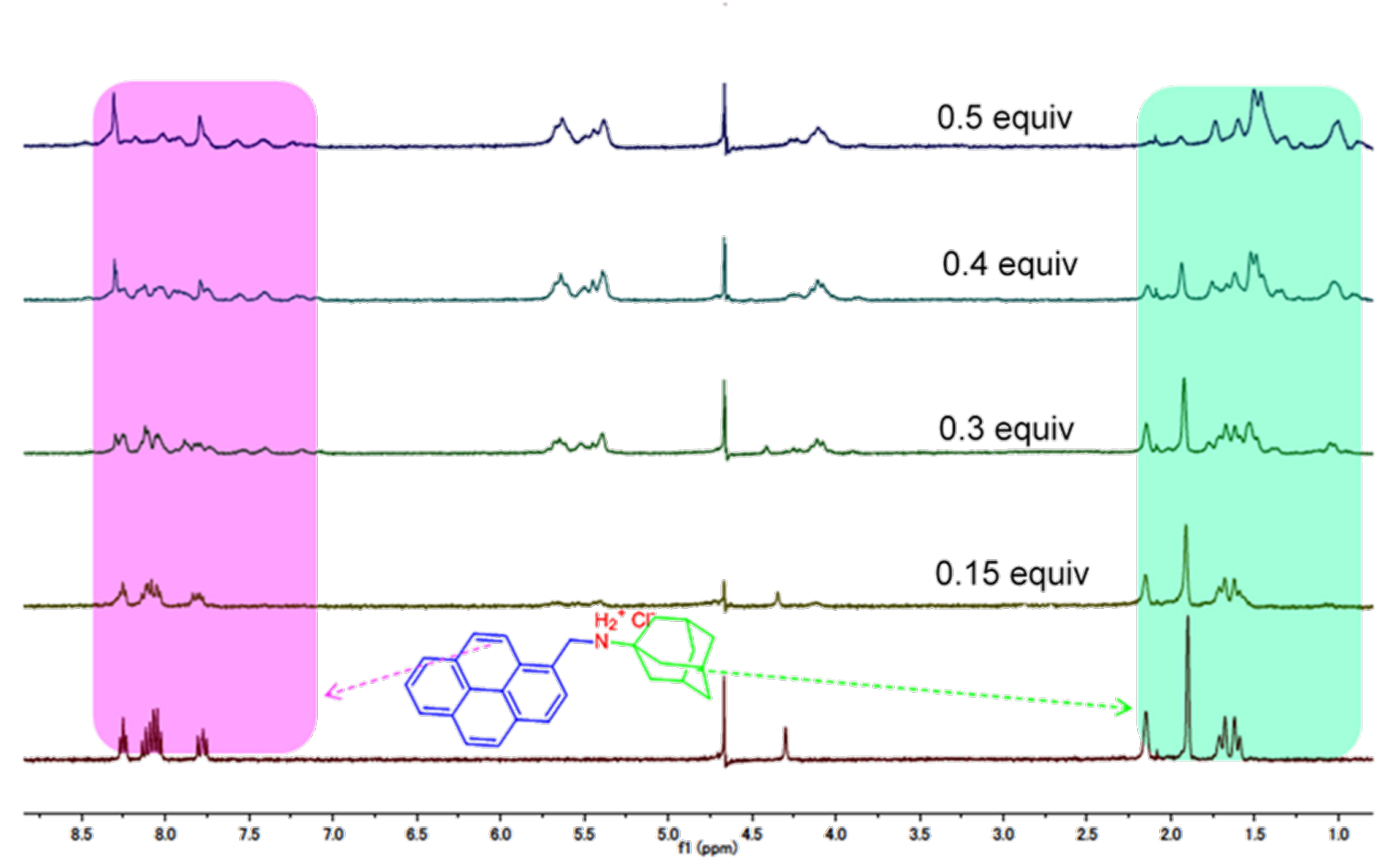
^1^H NMR spectra of **G2** (1.0 mmol, D_2_O, pD = 2.0) in the presence of different concentrations of host-**1**.

In order to obtain detailed information on the mechanism of the complexation of **G2** with host-**1** and -**2**, ^1^H NMR titration experiments in acidic aqueous solution were carried out. The spectral changes are shown in Figure 4. Upon addition of 0.5 equiv of host**-1** to the solution of **G2**, the chemical shift of some protons on the ADA moiety and pyrene group shifted upfield. Expectably, the upfield shift of ADA protons can be ascribed to the cavity’s encapsulation of this moiety. Pyrene group, that are too large for the individual CB[6]–CB[7] sized cavities of ns-CB[10] [23], it is thus believed that the upfield shift of the pyrene protons is attributed the intermolecular π–π stacking between the two pyrenyl moieties as proposed in the plausible inclusion modes in Scheme 2. Importantly, the ^1^H NMR spectral changes indicate that the rigid cavities of host-**2** can accommodate the ADA moiety of **G2** (Supporting Information File 1, Figure S5). The upfield shift of protons on the ADA moiety can be observed despite the fast formation of a precipitate upon the addition of host-**2** to a **G2** solution. This result is very different from our previous observations on the host–guest interactions of **G1** with host-**2** and those of a study on ADA with host-**2** by Isaacs and co-workers. In both cases, the ADA moiety was always rejected by the cavity of host-**2**.

Generally, the recognition binding ability for Q[*n*]s in aqueous solution is mainly due to the attraction between Q[*n*] hosts and guest based on the size or shape complementarily. This ability is aided by ion–dipole and dipole–dipole interactions arising from the electron-rich carbonyl rims of Q[*n*]s, the nonclassical hydrophobic effect of Q[*n*]s, and the classical hydrophobic effect of the guest [31–33]. The desolvation of the Q[*n*] host cavity delivers high-energy water trapped in the cavity; as it has a favorable enthalpic signature, this is a nonclassical hydrophobic effect. Contrary to that, the desolvation of the guest molecules is a classical hydrophobic effect; it has a favorable entropic component due to the delivery of surface-bound solvent molecules on the guest. Therefore, isothermal titration calorimetry could be employed to quantify the enthalpic and entropic contributions to the binding interactions between the hosts and guests (results are shown in Table 1 and Figures S6 and S7 in Supporting Information File 1). The obtained thermodynamic parameters suggest that the ternary host–guest interactions of host-**1** with **G1** and **G2**, host-**2** with **G1** are almost exclusively enthalpy-driven (Δ*H* = −50.90 kJ·mol^−1^ and *T*Δ*S* = −21.66 kJ·mol^−1^ for inclusion complex **G1·**host**-1**; Δ*H* = −65.77 kJ·mol^−1^ and *T*Δ*S* = −26.46 kJ·mol^−1^ for the inclusion complex **G2·**host**-1**; Δ*H* = −39.01 kJ·mol^−1^ and *T*Δ*S* = −1.02 kJ·mol^−1^ for the inclusion complex **G1·**host**-2**); while only the formation of the inclusion complexes of host**-2** with **G2** is driven by both enthalpy and entropy (Δ*H* = −30.96 kJ·mol^−1^ and *T*Δ*S* = 9.46 kJ·mol^−1^). Evidently, the high enthalpy gain for host-**1** with **G1** and **G2,** host-**2** with **G1** may be attributed to the strong ion–dipole interactions between the guest and host. However, the host–guest interactions in the case of complexes of **G2·**host-**2** are derived not only from the ion–dipole interactions between the host and guest, but also in the assistant of the hydrophobic effect. Notably, the entropic gain achieved with the **G2·**host-**2** system was higher than that obtained with other host–guest systems, indicating that the large hydrophobic side group pyrene is enabling the classical hydrophobic effect in aqueous solution as the guest indeed. On the other hand, the thermodynamic parameters obtained in the present study also suggest that the rigidify cavity structure such as host-**2** is benefit for the classical hydrophobic effect of guest in aqueous solution.

**Table 1:** Thermodynamic binding data for **G1**·host-**1**, **G2**·host-**1**, **G1**·host-**2**, and **G2**·host-**2** (error = ±10%).

Guest–host	*n*	*K*_a_ (L·mol^−1^)	Δ*G* (kJ·mol^−1^)	Δ*H* (kJ·mol^−1^)	*T*Δ*S* (kJ·mol^−1^)

**G1·**host**-1**	1.92	1.32 × 10^5^	−29.24	−50.90	−21.66
**G2·**host**-1**	1.98	7.64 × 10^6^	−39.3	−65.77	−26.46
**G1·**host**-2**	2.03	4.51 × 10^6^	−37.99	−39.01	−1.02
**G2·**host**-2**	1.99	1.08 × 10^7^	−40.42	−30.96	9.46

## Conclusion

In summary, we evaluated the molecular recognition triggered by a novel host–guest interaction of NS-CB[10]-based host**-1** and host-**2**. The unique monomer and excimer fluorescence emissions of pyrene due to the two-cavities of the host–guest system were exploited to identify the possible diastereomers. The top–center isomer of the ternary complexes of **G2** with the hosts was eventually identified from the distinct photophysical signals of pyrene. Interestingly, we found that being a large hydrophobic side group on the guests, pyrene plays important roles in improving the guest recognition and the binding ability of the hosts. This study shows that gradually tuning the side groups of the guest molecules from hydrophilic to hydrophobic may provide new insights into the dependence of molecular recognition on the host cavity size in aqueous solution.

## Supporting Information

File 1 Experimental data, additional ^1^H NMR spectra and others.
