# Supplementary material for: Host–guest interactions in nor-seco-cucurbit[10]uril: novel guest-dependent molecular recognition and stereoisomerism
Source: Beilstein J Org Chem. 2019 Jul 19;15:1705–11. doi: 10.3762/bjoc.15.166 (PMC6664405; doi:10.3762/bjoc.15.166)
Supplement: File 1 — Experimental data, additional 1H NMR spectra and others. [file Beilstein_J_Org_Chem-15-1705-s001.pdf]

BEILSTEIN JOURNAL OF ORGANIC CHEMISTRY

Supporting Information

for

Host–guest interactions in *nor*-*seco*-cucurbit[10]uril: novel guest-dependent molecular recognition and stereoisomerism

Xiaodong Zhang, Wei Wu, Zhu Tao and Xin-Long Ni

Beilstein J. Org. Chem. **2019,** *15,* 1705–1711. doi:10.3762/bjoc.15.166

Experimental data, additional ^{1}H NMR spectra and others

License and Terms: This is a supporting information file under the terms of the Creative Commons Attribution License (http://creativecommons.org/licenses/by/4.0). Please note that the reuse, redistribution and reproduction in particular requires that the authors and source are credited.
The license is subject to the *Beilstein Journal of Organic Chemistry* terms and conditions: (https://www.beilstein-journals.org/bjoc)

Synthesis of **G1**: 1-adamantanamine hydrochloride (1.87 g, 10.0 mmol) was dissolved in 30 ml ether, and an aqueous solution (10 ml) of 2.75 M NaOH was added into the ether solution with magnetic stirring for 30 minutes and then separated out the organic phase. Next, benzaldehyde (1.23 ml, 12 mmol), *p*-toluenesulfonic acid (0.1 g, 0.58 mmol) were added into the organic phase with magnetic stirring at room temperature for 5 h, then removed the ether by rotary evaporator. The white solid was dissolved in 50 ml ethanol and $NaBH_4$ (1.0 g, 0.026 mol) was added under ice bath for 1h. The mixture was stirred at 75 ℃ for another 3 h and concentrated by rotary evaporator. The solid was dissolved in HCl (0.3 M, 200 ml), and extracted with ether (50 ml) in order to remove unreacted impurities. Then the water phase become alkalinity used an aqueous sodium hydroxid, and extracted with ether (150 ml), and then removed the organic phase by rotary evaporator. The precipitation was recrystallized by HCl (2M, 10 ml), dried under vacuum to obtain a white solid 2.49 g (yield 89.8%). m.p. >250 °C. ^{1}H NMR (500 MHz, D_2O), δ 7.38-7.40 (5H, Ar-H) , 4.14 (2H, CH_2), 2.16 (3H, CH), 1.92 (6H, CH_2), 1.61-1.72 (6H, CH_2) ppm. ^{13}C NMR (125 MHz, d_6-DMSO): δ 131.86, 129.81, 129.17, 128.96, 57.79, 43.41, 37.98, 35.25, 29.32 ppm. MALDI-TOF: m/z calcd for $C_{17}H_{24}N^+$: 242.38; found: 243.27; elemental analysis calcd (%) for $C_{17}H_{24}N^+$: C 73.49, H 8.71, N 5.04; found: C 72.78, H 8.91, N, 4.96.

Synthesis of **G2**: A mixture of 1-adamantanamine hydrochloride (200 mg, 1.1 mmol) and sodium hydroxide (1.6 g, 0.04 mol) were heated at 40 °C in methanol (25 ml) with magnetic stirring for 1 h. The solution was recovered by filtration and the solution was transferred to flask, the 1-pyrenecarboxaldehyde (230 mg, 1 mmol) in 20 ml methanol was then added. The solution was refluxed for 12 h and cooled to room temperature. The cooled solution was added $NaBH_4$ (500 mg, 13.2 mmol) and then refluxed about 6 h and cooled to room temperature. The mixture was concentrated by rotary evaporator and the solid was dissolved in 60 ml CH_2Cl_2 and washed with 100 ml water three times. The organic layer was then concentrated by rotary evaporator and dissolved in 10 ml acetone solution accompany addition of small amount of HCl (1 M, 0.5 ml). The precipitation was collected under vacuum to obtain a yellow solid 258 mg (yield 64.2%). m.p. >250 °C .^{1}H NMR (500 MHz, D_2O), δ 7.67-8.33 (9H, Pyrene-H), 4.10 (2H, CH_2), 2.20 (3H, CH), 1.90 (6H, CH_2), 1.64-1.76 (6H, CH_2) ppm. ^{13}C NMR (125 MHz, CD_3OD-d_4): δ 132.46, 131.30, 130.63, 129.69, 128.87, 128.74, 128.35, 127.01, 126.38, 125.88, 125.67, 124.84, 124.75, 124.53, 124.20, 121.62, 58.25, 40.40, 38.07, 35.26, 29.39 ppm. MALDI-TOF: m/z calcd for $C_{27}H_{28}N^+$: 366.52; found: 366.91; elemental analysis calcd (%) for $C_{27}H_{28}NCl$: C 80.67, H 7.02, N 3.48; found: C 79.56, H 7.64, N, 3.31.

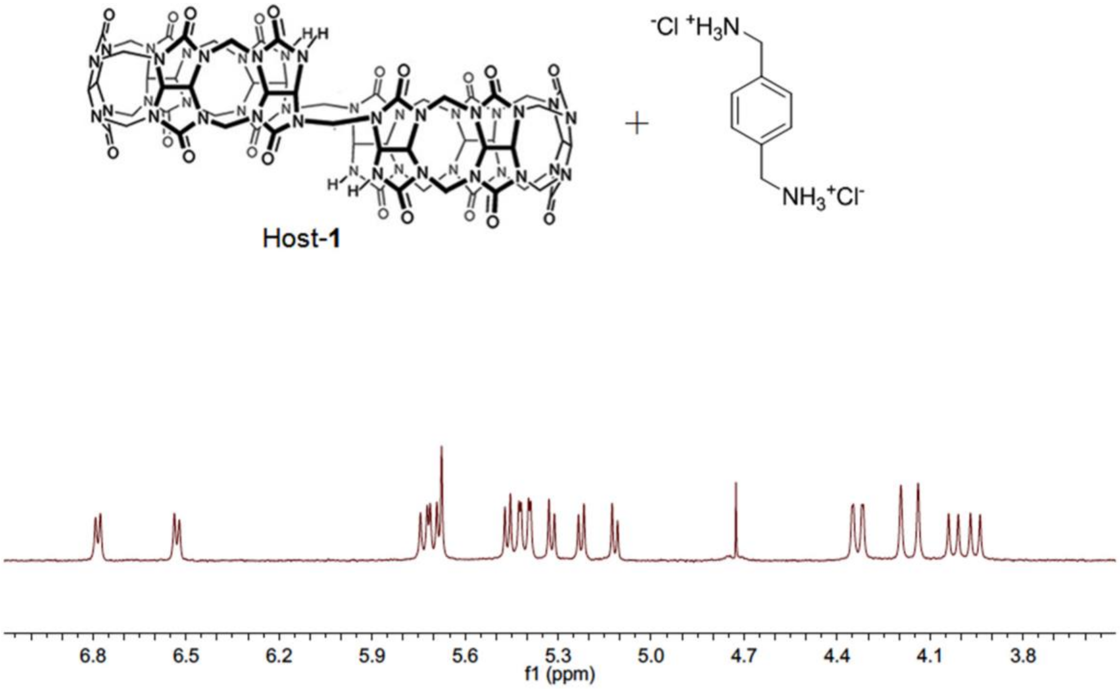

Figure S1 ^{1}H NMR spectra of host-**1** (1.0 mmol, D_2O, p*D* = 2.0) in the presence of *p*-xylylenediamine guest. The result is fully in line with the reference *J. Am. Chem. Soc.*, 2006, 128, 14744−14755.

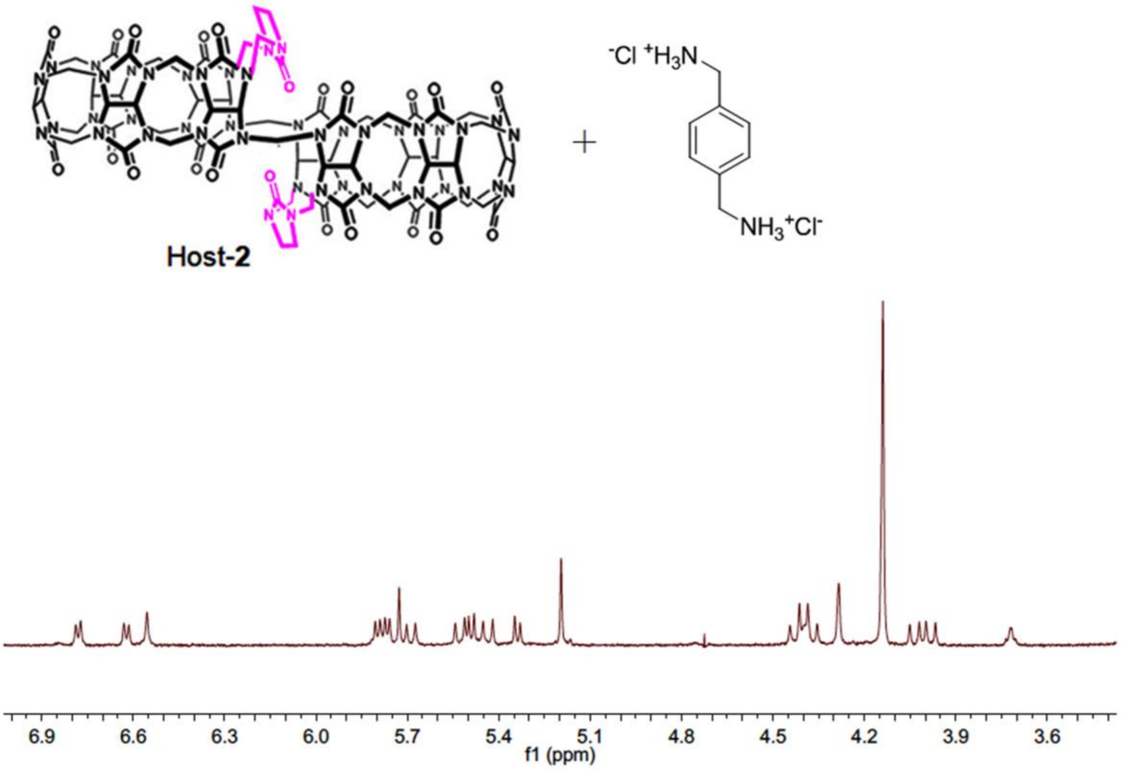

Figure S2 ^{1}H NMR spectra of host-**2** (1.0 mmol, D_2O, p*D* = 2.0) in the presence of *p*-xylylenediamine guest. The result is fully in line with the reference *Chem. Commun.*, 2011, 47, 9420–9422**.**

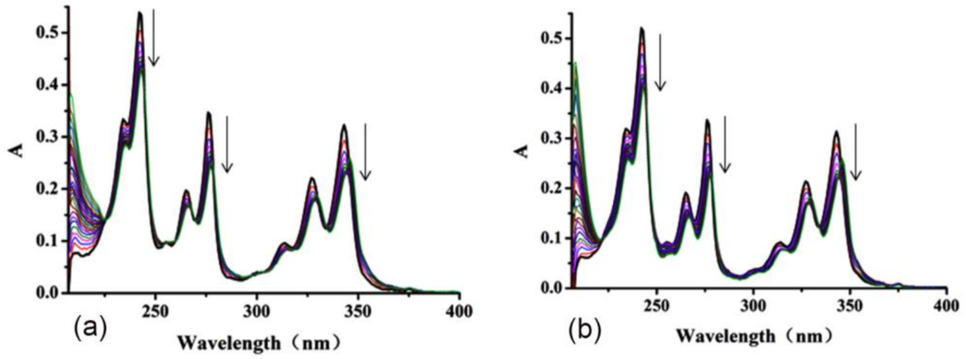

Figure S3 UV–vis spectral of **G2** (10.0 μM) in the presence of host-**1** (a) and host-**2** (b) at different concentrations (each of 30.0 μM) in aqueous solution (pH 2.0) at 298K.

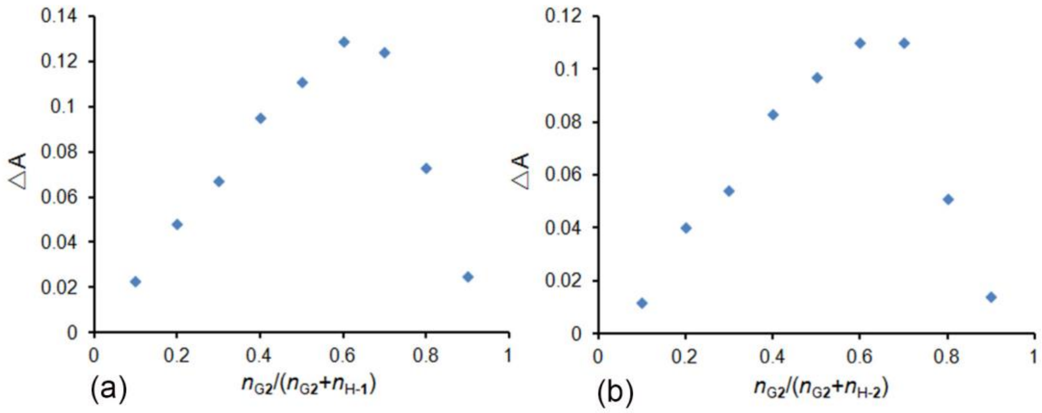

Figure S4 Job's plot showing the 2:1 binding of **G2** with host-**1** (a) and host-**2** (b).

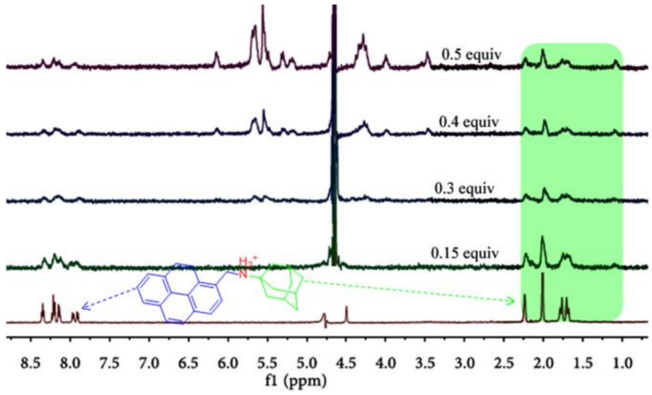

Figure S5 ^{1}H NMR spectra of G**2** (1.0 mmol, D_2O, p*D* = 2.0) in the presence of different concentrations of host-**2.**

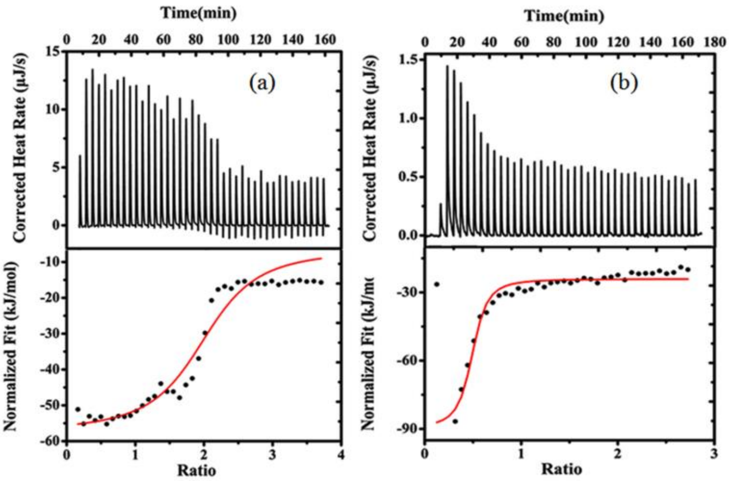

Figure S6 Isothermal titration calorimetry (ITC) binding curves for **G1**•host-**1** (a), and **G2**•host-**1** (b) in aqueous solution (pH 2.0) at 298.15 K (an independent model was used).

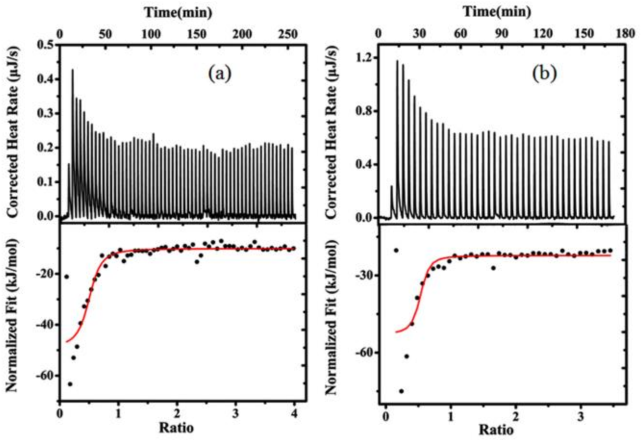

Figure S7 Isothermal titration calorimetry (ITC) binding curves for **G1**•host-**2** (a), and **G2**•host-**2** (b) in aqueous solution (pH 2.0) at 298.15 K (an independent model was used).

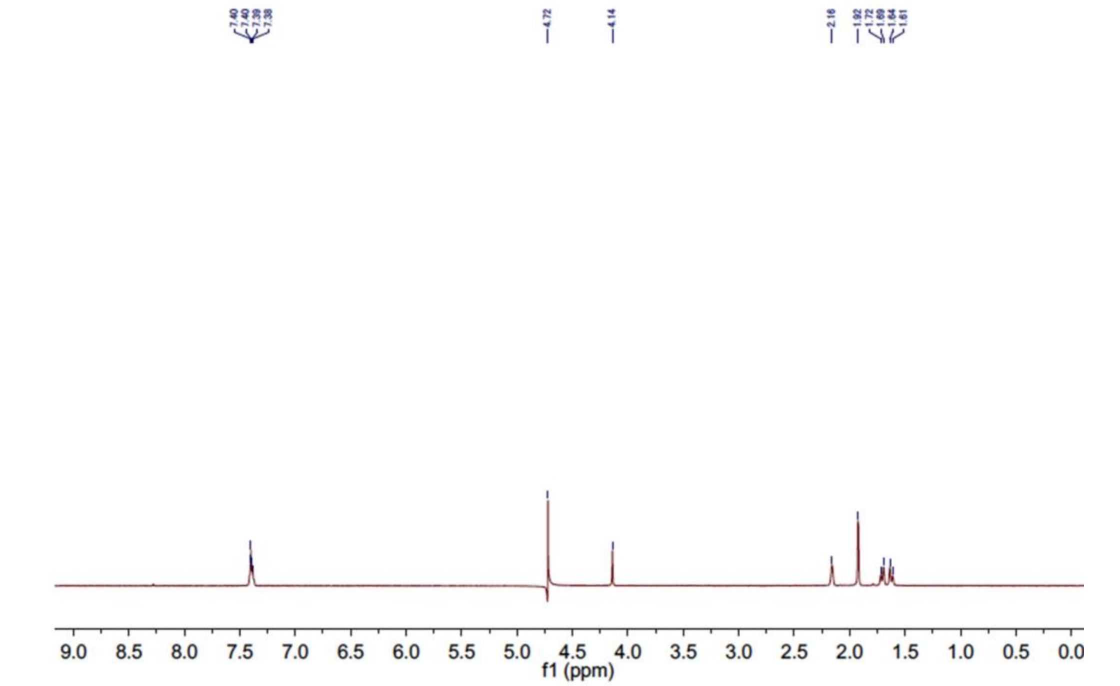

Figure S8 ^{1}H NMR spectra of **G1** (500 MHz, D_2O).

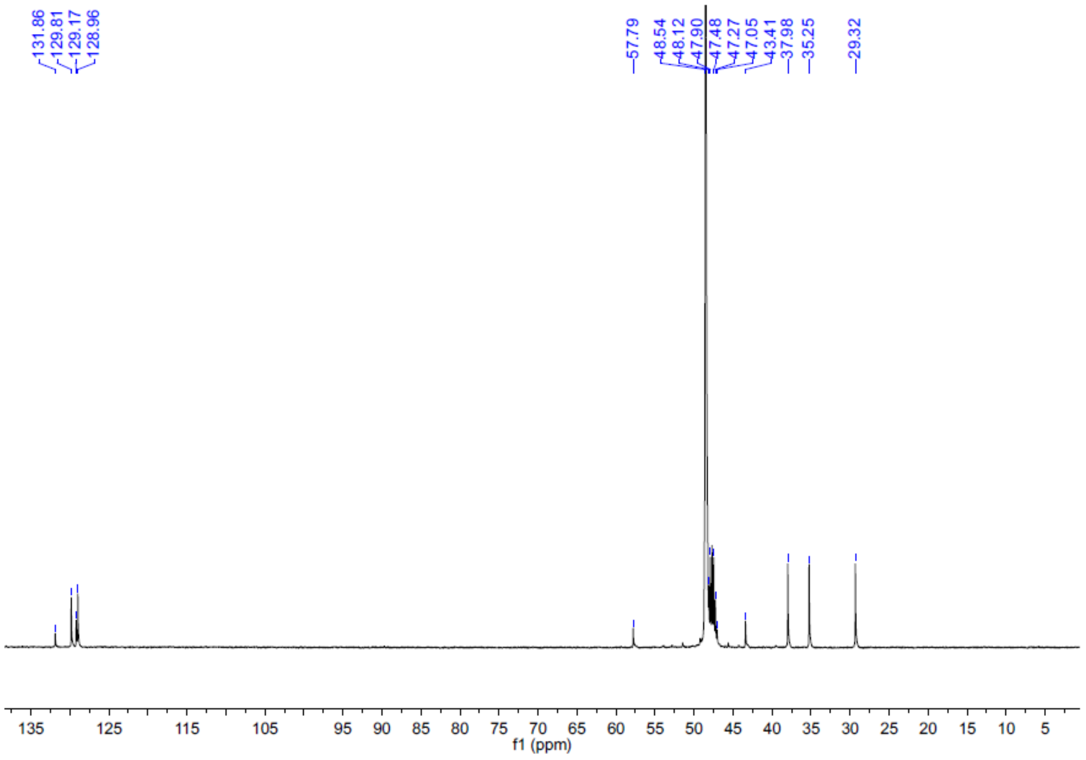

Figure S9 ^{13}C NMR spectra of **G1** (125 MHz, CD_3OD-d_4).

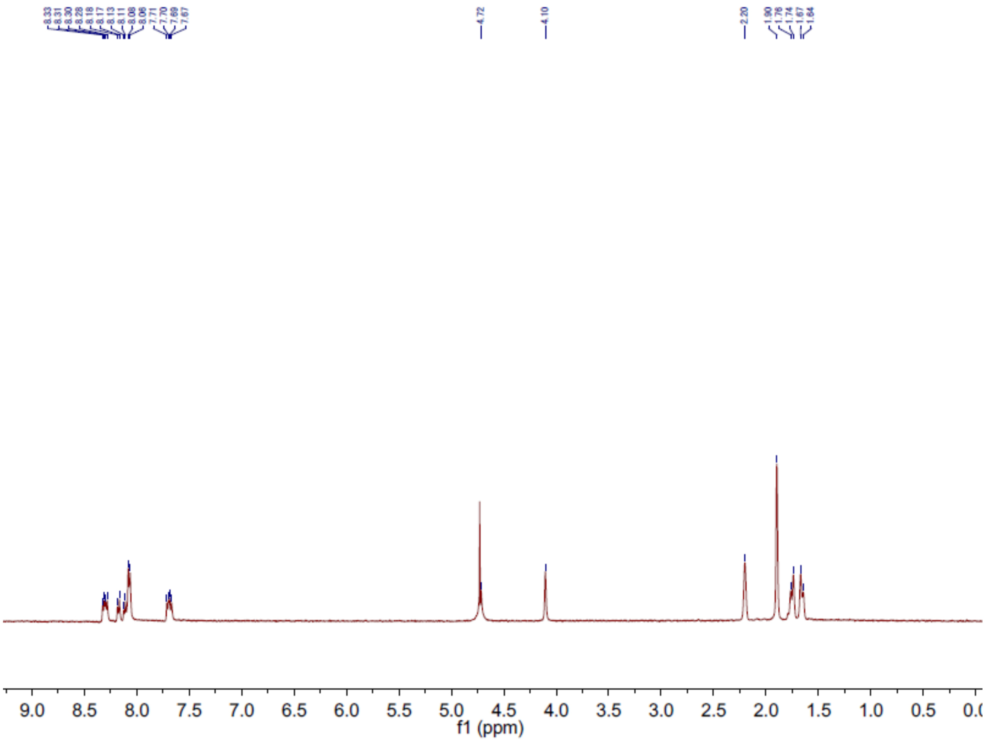

Figure S10 ^{1}H NMR spectra of **G2** (500 MHz, D_2O).

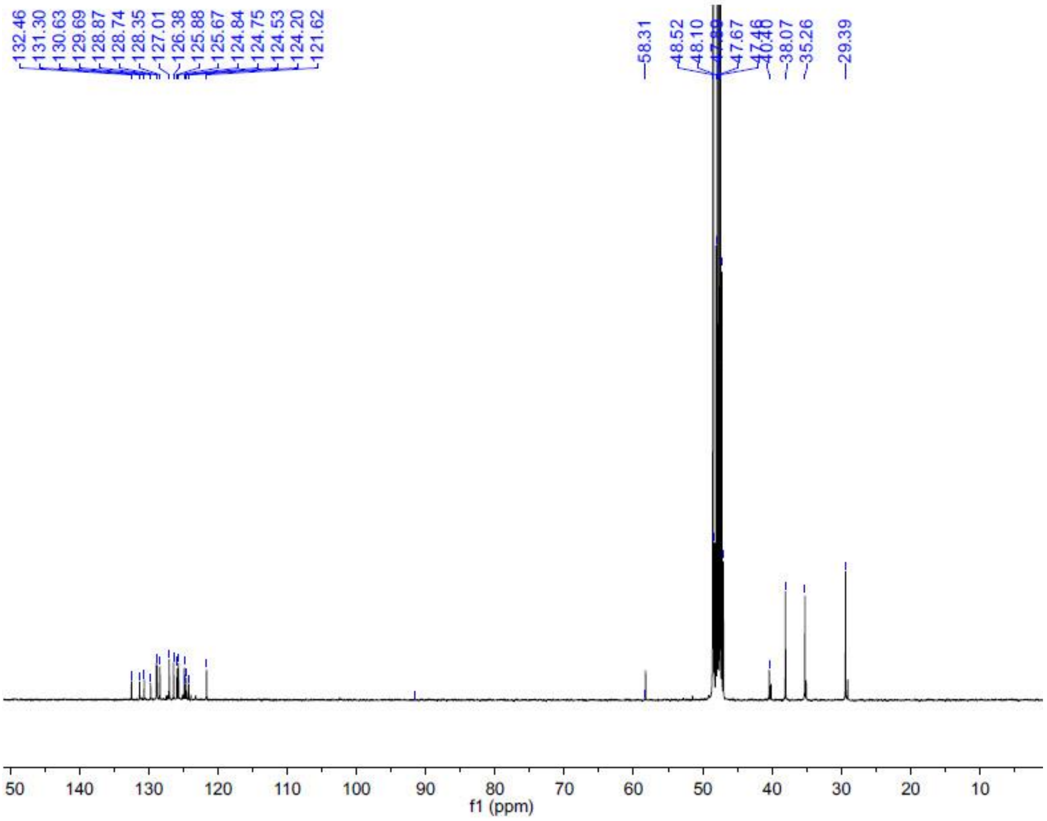

Figure S11 ^{13}C NMR spectra of **G2** (125 MHz, CD_3OD-d_4).
